# Graphene Oxide Functionalized Cottonseed-Lignin Resin with Enhanced Wet Adhesion for Woody Composites Application

**DOI:** 10.3390/polym14010001

**Published:** 2021-12-21

**Authors:** Zhiqiang Zhu, Erbing Zhang, Qinzhi Zeng, Jiuping Rao, Nairong Chen

**Affiliations:** College of Materials Engineering, Fujian Agriculture and Forestry University, Fuzhou 350002, China; mkkzq_zh@163.com (Z.Z.); 18726081410@163.com (E.Z.); fafurjp@163.com (J.R.)

**Keywords:** alkali lignin, graphene oxide, defatted cottonseed flour, adhesive, strength

## Abstract

With rising interior air pollution, health, and food shortage concerns, wood adhesives derived from non-food sustainable materials have therefore attracted considerable attention. Here we developed an eco-friendly cottonseed-lignin adhesive consisting of non-food defatted cottonseed flour (DCF), alkali lignin (AL), and graphene oxide (GO). The cation-π interaction, and hydrogen and covalent bonds between AL@GO and DCF collectively enhanced the cross-linking structure of the cured cottonseed-lignin adhesive, based on the Fourier-transform infrared spectroscopy, thermogravimetric analyses, scanning electron microscopy, and sol-gel tests. The high performance of the developed cottonseed-lignin adhesive was evidenced by its increased wet/dry shear strength and decreased rheological properties before curing and improved thermal stability and decreased soluble substances after curing. Particularly, the highest wet shear strength of poplar plywood bonded with cottonseed-lignin adhesive was 1.08 MPa, which increased by 74.2 and 54.3% as compared to the control and requirement of the Chinese standard GB/T 9846-2015 for interior plywood (≥0.7 MPa), respectively. The technology and resultant adhesives showed great potential in the preparation of green woody composites for many applications.

## 1. Introduction

Formaldehyde-based wood adhesives are currently widely used for making woody composites, which dominate most of the furniture consumption all over the world. However, formaldehyde is a known human carcinogen and non-renewable material [1]. Thus, the development of eco-friendly adhesives from sustainable biomass has attracted considerable attention in recent years. Many studies have been made on the use of soybean-based eco-friendly wood adhesives, and quite a number of positive results were obtained [2,3,4]. However, soybean is a kind of important food for humans and animals, and the industrial application of soybean-based adhesives might threaten the food supply. Therefore, it is necessary to develop a formaldehyde-free wood adhesive using non-food biomass as a promising alternative to soybean-based adhesives or formaldehyde-based adhesives.

Cotton is one of the most important fiber crops in the world. After the fiber is harvested, a large amount of cottonseed remains. Cottonseed is composed of shell, oil, and meal [5]. The product after shelling and degreasing is called cottonseed meal, which contains a chemical substance called gossypol that is toxic to humans and animals and is not considered as a food source for human consumption [6]. When cottonseed is mixed with another animal feed, its content cannot exceed the toxicity level tolerated by the animal. Currently, cottonseed and cottonseed meal are not fully utilized. Our previous work indicated that cottonseed meal is a suitable biomass for preparing wood adhesives with excellent water resistance when cross-linking to petroleum chemicals [7]. Hence, we are considering how to find the biomass-based cross-linker to develop a better eco-friendly adhesive, but the preparation of this adhesive has not been studied further. Lignin, an aromatic biomass composed of p-hydroxyphenyl, guaiacyl, and syringyl units, is characterized as being renewable, environmentally friendly, readily available, and low cost [8]. Due to the active functional groups, such as phenolic hydroxyl groups, alcoholic hydroxyl groups, and various modification sites in the lignin, it has been used as a substitute for phenol in adhesives [9,10]. Besides, the benzene rings in lignin provide a very stable structure for the resultant polymers, with excellent mechanical strength and thermal stability [11]. For example, lignin was added to polyurethane and epoxy resin adhesives to improve its oxidation resistance, thermal stability, and water resistance [12]. Moreover, it was reported that lignin is readily combined with protein to form a hydrophobic aggregation. For example, enzymatic hydrolysis of lignocellulosic biomass was accelerated in the solution containing soybean protein and protease as a result of lignin can form a strong intermolecular force with proteins [13,14]. Besides, when lignin was added to the soybean-based adhesive, it displayed performances on improved thermal stability or reduced the pores and cracks on the surface, and thus enhanced water resistance [15,16]. Therefore, lignin provides the possibility of an ideal biomass-based cross-linking agent for preparing cottonseed wood adhesives.

**Figure 1:**
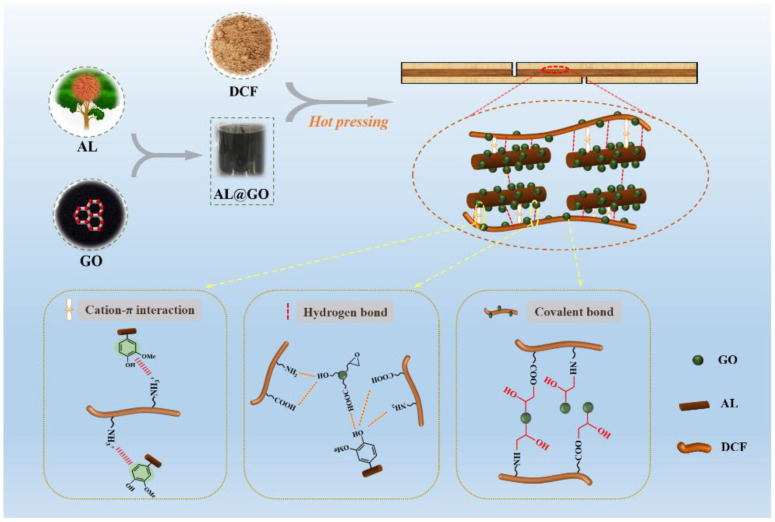
Schematic of the interaction of cottonseed-lignin adhesive components in woody composite materials.

Graphene, an atom of thick two-dimensional carbonaceous material with a hexagonal honeycomb crystal structure, has attracted great attention owing to its exceptional properties, such as excellent mechanical strength, and thermal and electronic conductivity. GO is the functionalized graphene via strong oxidants to chemically oxidize graphite. Thus, not only does GO have properties comparable with graphene, but also remarkable is the advantages of low production cost, large-scale production, and easy processing [17]. Moreover, GO contains numerous polarity functional groups, such as carbonyl, ester, lactone, epoxide, and hydroxyl, which endow GO to establish strong interfacial interactions between the filler and the polymeric matrix for composites with enhanced mechanical and hydrophobic properties [18]. Therefore, GO attracted a great deal of interest and has been widely used as a nanoreinforcement in polymer composite materials. Inspired by this, we used DCF as raw materials, and AL and GO as cross-linking agents to prepare an eco-friendly cottonseed-based adhesives. A possible pathway is shown schematically in Figure 1. AL has a large number of active functional groups which are readily combined with protein to form a hydrophobic aggregation, leading to improved water resistance of resultant adhesives. The addition of GO not only decreased viscosity in uncured adhesives by acting as a lubricant but also reacted to functional groups in adhesive components during the curing process. Hence, a 3-D structure was formed in the cured adhesives with excellent wet adhesion for woody composites application. Besides, the advantage of GO in wet adhesion performance was confirmed by its different usage; the interaction mechanism between DCF and the combination of AL and GO were confirmed by using Fourier-transform infrared spectroscopy (FTIR), thermogravimetric (TG), and scanning electron microscopy (SEM), sol-gel, rheological, and wet adhesion analyses. This technology provides an easy pathway to develop eco-friendly wood adhesive with excellent performance derived completely from renewable materials.

## 2. Materials and Methods

### 2.1. Materials

Defatted cottonseed flour, with pH 6.1, 50.0% of crude protein, 2.0% of fat, 7.5% of ash, 8.0% of moisture, and 98% of the mass of the flour passed a 300 mesh (0.150 mm) standard sieve, was purchased from Beijing Hongrun Baoshun Technology Co., Ltd. (Beijing, China). Alkali lignin (AL, average M_w_ ~10,000, 4% sulfur, pH = 10.5) was obtained from Beijing Soleibao Technology Co., Ltd. (Beijing, China). Multi-layer graphene oxide (GO, 95% purity, 45% of oxygen content, 5–10 of layers, 10–50 μm of lamellar diameter, ~1 nm in thickness), was purchased from Shenzhen Suiheng Technology Co., Ltd. (Shenzhen, China). The poplar veneer (300 mm × 300 mm in size, 1.7 mm in thickness, and 10–12% of moisture content) was purchased from Weide Wood Industry Co., Ltd. (Suzhou, China). Sodium hydroxide (NaOH) and other chemicals were analytical-grade reagents, and they were obtained from Sinopharm Chemical Reagent Co., Ltd. (Shanghai, China).

### 2.2. Adhesives Preparation

Mixtures containing 0, 0.01, 0.02, 0.03, or 0.04 g of GO in the 75.0 g deionized water were prepared via ultrasonic dispersion for 30 min at room temperature, respectively. Subsequently, one gram of AL was then added into each mixture and stirred at 35 °C for 30 min. Next, 25.0 g DCF was added to the mixture with containing stirring for 10 min. Then the pH of slurry was adjusted to 11.5 with 2 mol/L NaOH solution and further stirred for 30 min to obtain the GO functionalized cottonseed-lignin adhesives (DCF/AL@GO). In the control experiment, the mixture only contained 25.0 g DCF and 75.0 g deionized water. The adhesives with different content of GO were labeled DCF/AL, DCF/AL@GO-0.01, DCF/AL@GO-0.02, DCF/AL@GO-0.03, or DCF/AL@GO-0.04, respectively.

The adhesive samples were completely cured at 105 °C for 2 h in an oven. Then the prepared samples were stored in a desiccator for the subsequent analysis.

### 2.3. FTIR Analysis

FTIR analysis was carried out using a Nicolet 380 FTIR Spectrometer (Thermo Fisher Scientific, Waltham, MA, USA). The cured adhesive sample was mixed to potassium bromide, with mass ratio of 1:100, and then ground into a powder. About 100 mg of the powder was pressed into a tablet, which was scanned by the spectrometer at a range of 500–4000 cm^−1^ with a resolution of 4 cm^−1^ for 32 times. 

### 2.4. TG Analysis

TG analysis of adhesives was measured by a NETZSCH STA449C Synchronous Thermal Analyzer (NETZSCH Co., Selb, Germany). The cured adhesive (6.0 mg) was heated from 35 to 800 °C at a heating rate of 10 °C/min under nitrogen protection.

### 2.5. SEM Analysis

The fracture surface of the cured adhesive samples was coated with gold under vacuum and was observed via a Hitachi SU8010 scanning electron microscope (Tokyo, Japan).

### 2.6. Sol-Gel Analysis

A pre-weighed (m_1_) cured adhesive sample was mixed with deionized water at a weight ratio of 1:100, placed in a glass bottle, and kept at room temperature for 48 h. Then the sample in the glass bottle was washed and filtered via a filter paper with an average pore size of 3 nm. The residue on the filter paper was dried to constant weight at 103 °C and then weighed (m_2_). The sol fraction was calculated by the following equation: Sol fraction = (m_1_ − m_2_)/m_1_ × 100%. The results were recorded as the mean of triplicate.

### 2.7. Rheological Analysis

Rheological performance of the developed adhesives was measured using a Hacker rheometer (MARSIII, Thermo, Germany) at different shear rates (0~500 γ/s^−1^) using a PP35Ti parallel plate with distance of 0.105 mm. The shear stress and apparent viscosity were recorded in triplicate at room temperature.

### 2.8. Wet Adhesion Analysis

The cottonseed-based adhesive was applied in making three-layer poplar plywood. About 170 g/m^2^ of the adhesive was coated on one side of a veneer, which was cross laid up to another coated veneer by the coated side with uncoated side, and then the coated side was covered with an uncoated veneer. The assembled plywood was pressed using the following parameters: 130 °C hot pressing temperature, 1.2 MPa pressing pressure, and 5.0 min pressing time. The prepared plywood was stored at room temperature for 24 h before the shear strength test. 

**Figure 2:**
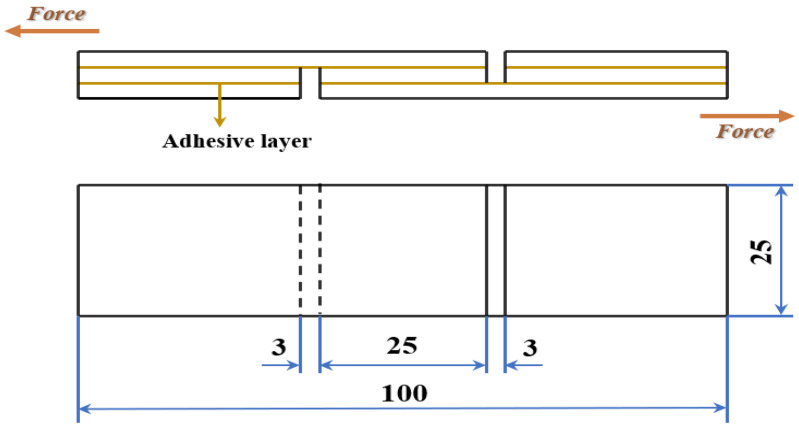
The diagram of shear strength test specimen (Units: mm).

According to the methods description in the Chinese National Standards GB/T 17657-2013, the wet adhesion performance of the developed adhesive was evaluated by shear strength of plywood after soaking treatment. Each plywood was cut into 10 pieces with dimension of 100 mm × 25 mm (Figure 2) and then the samples were placed in a 63 °C water bath for 3 h. After cooling at room temperature for 10 min, shear strength of plywood was recorded by using a universal testing machine (MTS, Shenzhen, China) with a crosshead speed of 10 mm/min. The result was adopted as the average of 10 samples in each group.

## 3. Results and Discussion

### 3.1. Characterization

**Figure 3:**
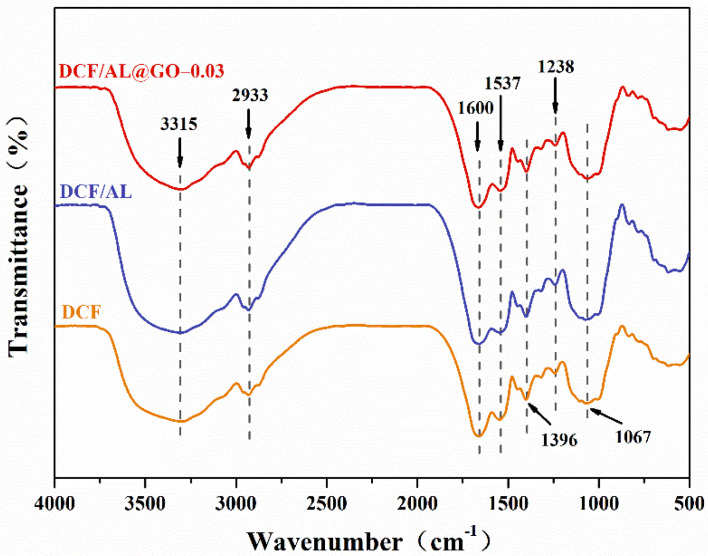
FTIR spectra of cottonseed-lignin adhesives with different cross-linker.

In order to further study the possible interaction of DCF, AL, and GO in cottonseed-based adhesives, FTIR spectroscopy analysis was performed. As shown in Figure 3, the spectrum of the control sample displays absorption peaks of the protein at 1660, 1537, and 1238 cm^−1^, which were characteristic of amide I (C = O stretching), amide II (N-H bending), and amide III (C-N and N-H stretching), respectively [19]. Besides, the absorption peaks at 3315 cm^−1^ are assigned to the bending vibration of the O-H and N-H groups, 2930 cm^−1^ are attributed to the symmetric and asymmetric tensile vibrations of the -CH_2_ groups, and 1052 cm^−1^ are assigned to the C-O bending in ether [2]. These peaks were showed in the spectra of DCF/AL and DCF/AL@GO-0.03. No new peak was detected for DCF modified with AL or GO indicating that: (i) the cross-linking took place without the formation of the chemical bonds mentioned before; (ii) due to the large number of chemical bonds already present in the cottonseed protein, it was not possible to detect the formation of any additional new bonds due to the overlap of several peaks in the fingerprint regions; and (iii) the combination between lignin and cottonseed protein was the cation-π interaction [14]. However, the effects of AL or GO on cottonseed-based adhesives could be readily confirmed using other characterization techniques, including thermal stability, sol-gel analysis, and wet adhesion test.

**Figure 4:**
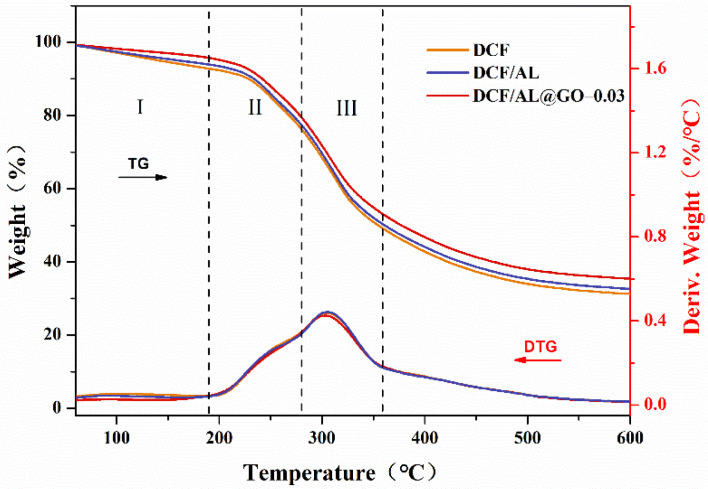
Thermal degradation curves of cottonseed-lignin adhesives with different cross-linker.

Figure 4 shows the thermogravimetric curve and derivative thermogravimetric (DTG) curve of cottonseed-lignin adhesives. The thermal degradation process of the adhesives could be divided into three stages [20]. The first stage (I) was due to the evaporation of residual moisture in the temperature range of 50–190 °C. The second stage (II) was the initial degradation stage from 190 to 280 °C, which was caused by the weight loss of small molecules to be degraded and the breaking of some unstable chemical bonds. The third stage (III) was the degradation stage of the framework structure with a temperature range of 280–360 °C, which was attributed to the degradation of the cross-linked network structure [21]. In the second stage, the weight loss of DCF/AL@GO-0.03 adhesive was about 15%, which was 1% lower than that of DCF or control adhesive. It was suggesting the better thermal stability in the DCF/AL@GO-0.03 adhesives. The tendency was more obvious in the third stage, which was the degradation of the adhesive backbone, including the breakdown of the peptide bonds of the cottonseed protein backbone and the cleavage of the C-O, S-S, and O-N bonds [22]. At this stage, the maximum degradation rate of DCF/AL@GO-0.03 adhesive was significantly lower than that of the DCF/AL and DCF adhesives. These indicate that the cross-linked structures of the cottonseed-based adhesive could be enhanced by adding AL and GO, thereby improving the thermal stability, water resistance, and mechanical properties of the resultant cottonseed-lignin adhesives [23].

**Figure 5:**
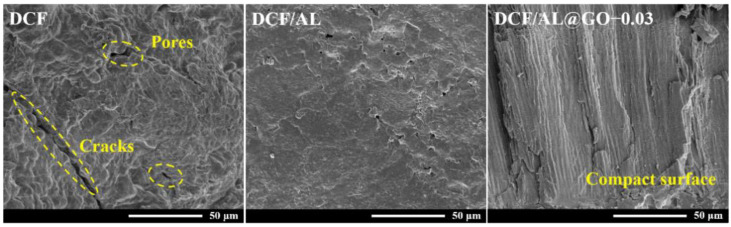
SEM images of cottonseed-lignin adhesives with different cross-linker.

The SEM image of different curing adhesives is shown in Figure 5. DCF or control sample had a loose multi-pore structure which made it easier for water to penetrate into the curing adhesive and caused the high water absorption and dissolution, leading to poor water resistance of the DCF adhesive [24,25]. The sample with AL (DCF/AL) had a smooth surface without large-area pores. AL was evenly dispersed in the adhesive and the dense and compact structure was observed. Generally, the smaller the pores of the curing adhesive, the better performance in the moisture resistance test due to the poor water absorption. In a comparison of DCF/AL@GO-0.03 and DCF/AL, the former displayed a smoother fracture surface, indicating that compact structure was generated in the curing process of the adhesives. The compact structure and smooth surface of the curing adhesives will more likely increase cross-linkage in cured adhesive and hence improve its mechanical strength and water resistance [26].

### 3.2. Sol-Gel Test

**Figure 6:**
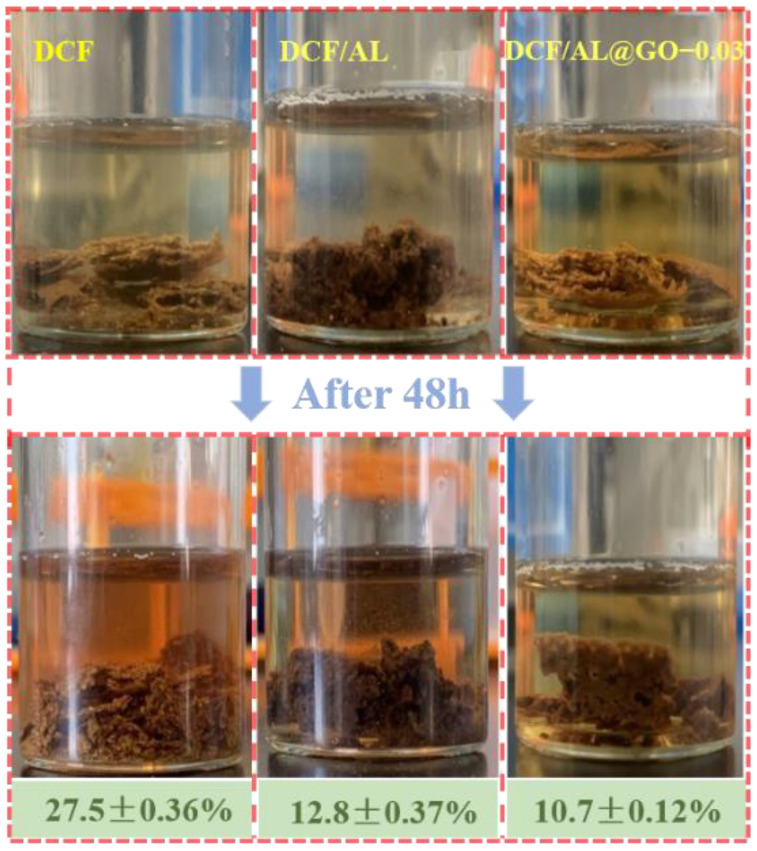
Sol-gel test of cured cottonseed-lignin adhesives with different cross-linkers.

The sol-gel test for cottonseed-lignin adhesives showed that the DCF or control sample partially disintegrated in water with a turbid condition, while the DCF/AL or DCF/AL@GO-0.03 remained intact after being soaked in a water bath at room temperature for 48 h (Figure 6). This indicated that the cross-linked structure in the control sample was lower than the modified sample [27]. As evidenced by the results, the gel fraction (cross-linked part) of the control and DCF/AL were calculated to be 72.5 and 87.2%, respectively. However, the gel fraction of the DCF/AL was lower than the DCF/AL@GO-0.03, suggesting that the later adhesive sample with GO displays the better cross-linked structure, which significantly enhances the water resistance of the bonded plywood.

### 3.3. Rheology Properties

**Figure 7:**
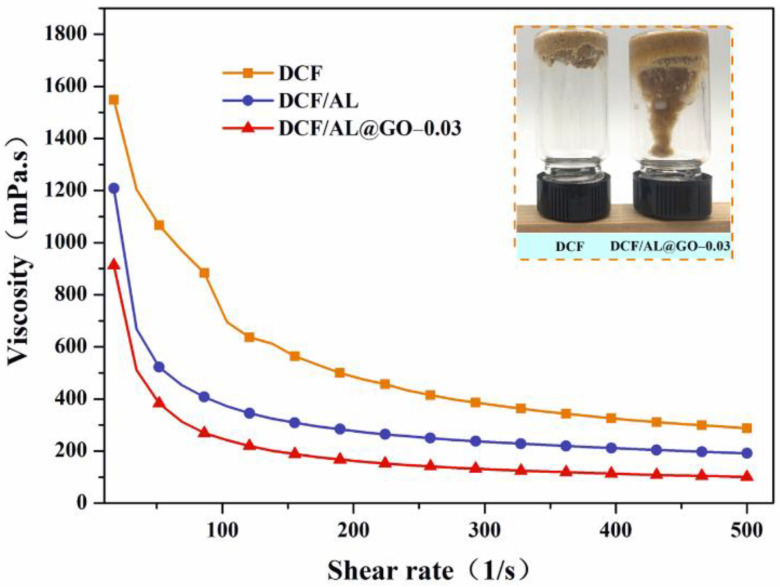
Rheological curves of cottonseed-lignin adhesive with different cross-linkers.

Viscosity of adhesive is a great influence on its operability in the application process. Figure 7 shows the change in the viscosity under different shear rates. The Newtonian fluid or shear-thinning behavior was observed in all adhesives due to the viscosity of all adhesives gradually decreasing with the increase in the shear rate [28]. This phenomenon will benefit the rolling application of the wood adhesives because the adhesive spreaders roll with fast rotational speed [29]. Comparing the viscosity of the adhesives under the consistent shear rate shows that the viscosity was in the order of the control > DCF/AL > DCF/AL@GO-0.03. This indicates that the viscosity of adhesives could be decreased by the AL, but the combination of AL and GO shows better effects for decreased viscosity. Generally, low viscosity will facilitate the adhesive penetrating into the crack or pore of wood and the further formation of cured adhesive-wood mechanical interlocking, which greatly enhances mechanical properties of the resultant composites [30].

### 3.4. Adhesiveness

**Figure 8:**
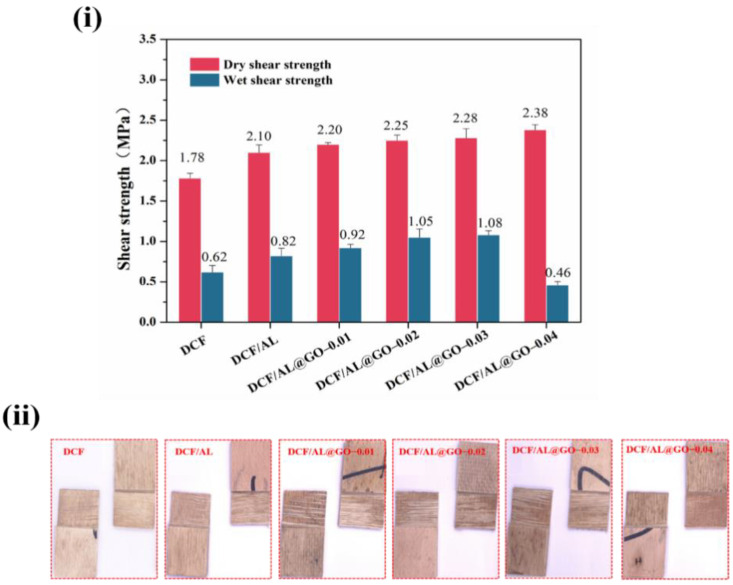
Adhesion performance of cottonseed-lignin adhesives evaluated by (**i**) shear strength and (**ii**) wood failure of plywood.

Wet adhesion is essential for the practical application of an adhesive in the wood-based composite. Figure 8 shows the dry and wet shear strength of plywood bonded with cottonseed-lignin adhesives. After being soaked in a 63 °C water bath for 3 h, plywood bonded with the control had the lowest wet shear strength (only 0.62 MPa), which does not meet the requirement of the Chinese national standard GB/T 9846-2015 for interior plywood (≥0.7 MPa). It suggests that the pure DCF resin displayed a weak wet adhesion performance due to the hydrophilic properties of cottonseed protein, which is easily destroyed in a moist environment [31]. After the introduction of the AL, the adhesive (DCF/AL) showed an improved wet adhesion with the wet shear strength of 0.82 MPa, which is mainly due to the cation-π interaction between the cationic group in DCF protein (R-NH_3_^+^) and the aromatic π-electron in AL. The cation-π interaction significantly enhanced the cross-linking density of the DCF/AL composite [14]. Besides, the introduction of AL also improved the coating performance which promotes the binding of adhesive and wood substrate. However, it has limits to the effect of reducing viscosity. The addition of GO not only greatly reduced the viscosity of adhesive but further improved the wet adhesion of plywood. The results agree with the sol-gel test analysis. We speculate that the increased cross-linking structure was formed in the curing adhesive with a combination of AL and GO. The wet shear strength increased as the amount of GO in adhesives was increased and then decreased at the GO usage of about 0.3% and beyond. Comparing to the DCF or control, the highest wet shear strength of DCF/AL@GO-0.03 was increased by 74.2% to 1.08 MPa and with improved wood failure (Figure 8). Similar results were observed in the dry shear strength and wood failure (Figure 8) of plywood. This is ascribed to three factors (Figure 1): (i) the lubrication of GO further reduces the viscosity of adhesive, which was readily penetrated into the wood substrate and formed the mechanical interlocking structure; (ii) the functional groups of DCF and AL can form hydrogen bonds with carbonyl and hydroxyl groups of GO, which enhanced the mechanical properties of adhesives; and (iii) the epoxy group of GO cross-linked with the functional groups of DCF (-NH_2_, -COOH) to consume the hydrophilic groups [32,33]. The reactions are expected to form a three-dimensional cross-linked structure that significantly improves the wet adhesion of the adhesives. Particularly, comparing to the requirements of GB/T 9846-2015, the wet shear strength (1.08 MPa) increased by 54.3%, showing great potential for woody composite panels in practical application.

## 4. Conclusions

Using a combination of AL and GO as a biomass-based cross-linking agent to effect cross-link DCF, sustainable, eco-friendly cottonseed-lignin adhesives were developed and evaluated in this work. The wet adhesion performance was improved significantly by using AL and enhanced further by combining AL with GO. The synergistic effects of improved bonding strength, moisture resistance, and thermal stability of cottonseed-lignin adhesives as a result of the cation-π interaction, hydrogen bonds, and covalent bonds between DCF components and AL@GO contributed to the higher adhesion performance. The eco-friendly adhesive derived from non-food renewable materials has great potential as a substitute for traditional formaldehyde-based wood adhesives.

## Data Availability

Not applicable.

## References

[1] Li J., Pradyawong S., He Z.Q., Sun X.Z.S., Wang D.H., Cheng H.N., Zhong J.Y. (2019). Assessment and application of phosphorus/calcium-cottonseed protein adhesive for plywood production. J. Clean. Prod..

[2] Xu C.J., Xu Y.C., Chen M.S., Zhang Y., Li J.Z., Gao Q., Shi S.Q. (2020). Soy protein adhesive with bio-based epoxidized daidzein for high strength and mildew resistance. Chem. Eng. J..

[3] Chen N., Huang J., Li K.J.H. (2018). Investigation of a new formaldehyde-free adhesive consisting of soybean flour and Kymene^®^ 736 for interior plywood. Holzforschung.

[4] Zhao S., Wang Z., Li Z., Li L., Li J., Zhang S. (2019). Core-Shell Nanohybrid Elastomer Based on Co-Deposition Strategy to Improve Performance of Soy Protein Adhesive. ACS Appl. Mater. Interfaces.

[5] Cheng H.N., Dowd M.K., He Z.Q. (2013). Investigation of modified cottonseed protein adhesives for wood composites. Ind. Crops Prod..

[6] He Z.Q., Hailin Z., Olk D.C., Mark S., Way T.R., Haile T.J. (2014). Protein and Fiber Profiles of Cottonseed from Upland Cotton with Different Fertilizations. Mod. Appl. Sci..

[7] Chen N.R., Huang J., Li K.C. (2020). Investigation of a Formaldehyde-free Cottonseed Flour-based Adhesive for Interior Plywood. Bioresources.

[8] Zhao S., Abu-Omar M.M. (2017). Synthesis of Renewable Thermoset Polymers through Successive Lignin Modification using Lignin-Derived Phenols (LDPs). ACS Sustain. Chem. Eng..

[9] Luo J., Yuan C., Zhang W., Li J., Gao Q. (2015). An eco-friendly wood adhesive from soy protein and lignin: Performance properties. RSC Adv..

[10] Chen S.L., Wang G.H., Sui W.J., Parvez A.M., Si C.L. (2020). Synthesis of lignin-functionalized phenolic nanosphere supported Ag nanoparticles with excellent dispersion stability and catalytic performance. Green Chem..

[11] Zhang Y., Wu J.Q., Li H., Yuan T.Q., Wang Y.Y., Sun R.C. (2017). Heat Treatment of Industrial Alkaline Lignin and its Potential Application as an Adhesive for Green Wood-Lignin Composites. ACS Sustain. Chem. Eng..

[12] Gioia C., Lo Re G., Lawoko M., Berglund L. (2018). Tunable Thermosetting Epoxies Based on Fractionated and Well-Characterized Lignins. J. Am. Chem. Soc..

[13] Luo X., Liu J., Zheng P., Li M., Zhou Y., Huang L., Chen L., Shuai L. (2019). Promoting enzymatic hydrolysis of lignocellulosic biomass by inexpensive soy protein. Biotechnol. Biofuels.

[14] Zheng P., Xiang L., Chang J., Lin Q., Xie L., Lan T., Liu J., Gong Z., Tang T., Shuai L. (2021). Nanomechanics of Lignin-Cellulase Interactions in Aqueous Solutions. Biomacromolecules.

[15] Xiao Z.G., Li Y.H., Wu X.R., Qi G.Y., Li N.B., Zhang K., Wang D.H., Sun X.Z.S. (2013). Utilization of sorghum lignin to improve adhesion strength of soy protein adhesives on wood veneer. Ind. Crops Prod..

[16] Wang W., Xu G. (2007). Effects of Soy-adhesive/PF Mixture on Shear Strength of Plywood. J. Northeast For. Univ..

[17] Dreyer D.R., Park S., Bielawski C.W., Ruoff R.S. (2010). The chemistry of graphene oxide. Chem. Soc. Rev..

[18] Agarwal V., Zetterlund P.J. (2020). Strategies for reduction of graphene oxide—A comprehensive review. Chem. Eng. J..

[19] Gu W.D., Liu X.R., Li F., Shi S.Q., Li J.Z. (2019). Tough, strong, and biodegradable composite film with excellent UV barrier performance comprising soy protein isolate, hyperbranched polyester, and cardanol derivative. Green Chem..

[20] Li J.J., Luo J., Li X.N., Yi Z., Gao Q., Li J.Z. (2015). Soybean meal-based wood adhesive enhanced by ethylene glycol diglycidyl ether and diethylenetriamine. Ind. Crops Prod..

[21] Qi G., Sun X.S. (2011). Soy Protein Adhesive Blends with Synthetic Latex on Wood Veneer. J. Am. Oil Chem. Soc..

[22] Yi Z., Meng Z., Chen M., Jing L., Li X., Qiang G., Li J.J. (2018). Preparation and characterization of a soy protein–based high-performance adhesive with a hyperbranched cross-linked structure. Chem. Eng. J..

[23] Yin H., Zhang E.B., Zhu Z.Q., Han L.B., Zheng P.T., Zeng H.B., Chen N.R. (2021). Soy-Based Adhesives Functionalized with Pressure-Responsive Crosslinker Microcapsules for Enhanced Wet Adhesion. ACS Appl. Polym. Mater..

[24] Chen N.R., Lin Q.J., Rao J.P., Zeng Q.Z. (2013). Water resistances and bonding strengths of soy-based adhesives containing different carbohydrates. Ind. Crops Prod..

[25] Ghosh Dastidar T., Netravali A.N. (2013). A soy flour based thermoset resin without the use of any external crosslinker. Green Chem..

[26] Chen N.R., Zeng Q.Z., Lin Q.J., Rao J.P. (2015). Development of defatted soy flour based bio-adhesives using Viscozyme L.. Ind. Crops Prod..

[27] Zeng Y., Xu P., Yang W., Chu H., Ma P. (2020). Soy protein-based adhesive with superior bonding strength and water resistance by designing densely crosslinking networks. Eur. Polym. J..

[28] Arshad M., Maali A., Claudet C., Lobry L., Peters F., Lemaire E. (2021). An experimental study on the role of inter-particle friction in the shear-thinning behavior of non-Brownian suspensions. Soft Matter.

[29] Yin H., Zheng P.T., Zhang E.B., Rao J.P., Lin Q.J., Fan M.Z., Zhu Z.Q., Chen M.Q., Cheng S.Y., Zeng Q.Z. (2021). An environmentally-friendly soybean based resin as an alternative to formaldehyde-based counterpart for biomass composites. Int. J. Adhes. Adhes..

[30] Zheng P., Chen N., Islam S., Ju L.K., Liu J., Zhou J., Chen L., Zeng H., Lin Q.J. (2018). Development of Self-Cross-Linked Soy Adhesive by Enzyme Complex from Aspergillus niger for Production of All-Biomass Composite Materials. ACS Sustain. Chem. Eng..

[31] Zhang Y., Shi R., Xu Y., Chen M., Zhang J., Gao Q., Li J. (2019). Developing a stable high-performance soybean meal-based adhesive using a simple high-pressure homogenization technology. J. Clean. Prod..

[32] Luo J., Zhou Y., Gao Q., Li J.Z., Yan N. (2020). From Wastes to Functions: A New Soybean Meal and Bark-Based Adhesive. ACS Sustain. Chem. Eng..

[33] Berman D., Erdemir A., Sumant A.V. (2013). Few layer graphene to reduce wear and friction on sliding steel surfaces. Carbon.

